# Evodiamine Alleviates 2,4-Dinitro-1-Chloro-Benzene-Induced Atopic Dermatitis-like Symptoms in BALB/c Mice

**DOI:** 10.3390/life14040494

**Published:** 2024-04-11

**Authors:** So-Young Han, Dong-Soon Im

**Affiliations:** Department of Fundamental Pharmaceutical Sciences, Graduate School, Kyung Hee University, Seoul 02447, Republic of Korea; wlsruddk62@khu.ac.kr

**Keywords:** *Evodia rutaecarpa*, atopy, evodiamine, dermatitis, eczema, anti-atopy

## Abstract

Evodiamine is an alkaloid found in Evodia fruits, a traditional Chinese medicine. Preclinical studies have demonstrated its anti-inflammatory and neuroprotective properties. The 2,4-dinitro-1-chloro-benzene (DNCB) was used to test the effects of evodiamine on a chemically induced atopic dermatitis-like model in BALB/c mice. Evodiamine significantly lowered serum immunoglobulin E levels, which increased as an immune response to the long-term application of DNCB. Several atopic dermatitis-like skin symptoms induced by DNCB, including skin thickening and mast cell accumulation, were suppressed by evodiamine therapy. DNCB induced higher levels of pro-inflammatory cytokines in type 2 helper T (Th2) cells (IL-4 and IL-13), Th1 cells (IFN-γ and IL-12A), Th17 cells (IL-17A), Th22 cells (IL-22), and chemokines (IL-6 and IL-8). These increases were suppressed in the lymph nodes and skin following evodiamine treatment. The results of our study indicate that evodiamine suppresses atopic dermatitis-like responses in mice and may therefore be useful in treating these conditions.

## 1. Introduction

Chronic atopic dermatitis is an inflammatory, pruritic skin condition characterized by persistent skin inflammation that often occurs in families with other atopic diseases [1,2]. Various factors, including defects in skin barrier function due to structural protein formation such as filaggrin, abnormal lipid metabolism, environmental factors, and genetic factors, contribute to dysregulated immune responses [1,3]. Pediatric atopic dermatitis patients comprise the majority (1–3% of adults and up to 20% of children) in most countries [4,5]. A hallmark of atopic dermatitis is the presence of widespread eczematous lesions [6]. Atopic dermatitis is often the first step in the development of other atopic diseases, such as allergic rhinoconjunctivitis and/or bronchial asthma [7]. Although elevation of total or allergen-specific immunoglobulin E (IgE) levels in the serum is not always observed in all individuals, atopic dermatitis is characterized by type 2 immune responses and consequently increased IgE levels [8,9]. In addition to the Th2 response, the Th1 and Th17 responses also play key roles in pathogenesis [10,11,12].

Current therapeutics include glucocorticoids as a first-line anti-inflammatory treatment, topically applied to the skin for pruritus or new flares [5]. In adults, topical application of tacrolimus and pimecrolimus is permitted for eczema treatment [13]. Since mast cell mediators, such as tryptase and histamine, play important roles in pruritus induction in atopic dermatitis, the application of mast cell stabilizers has also been considered [14]. However, owing to the side effects of long-term glucocorticoid use, alternative therapeutics must be developed [15]. Traditional Chinese medicine can provide a variety of resources for the development of new drugs for atopic dermatitis [16].

The dried, unripe fruit of *Evodia rutaecarpa* Bentham (Rutaceae) has been used to treat epilepsy, emesis, dermatophytosis, dysentery, gastrointestinal disorders, and headaches [17]. As alkaloidal compounds found in Evodia fruits, evodiamine and rutaecarpine have shown a number of pharmacological properties in preclinical models, including anti-inflammatory, anti-obesity, anti-bacterial, anti-cancer, anti-cardiovascular, anti-diabetic, insecticide, and neuroprotective effects [17]. Shin et al. found that evodiamine and rutaecarpine inhibit the production of pro-inflammatory cytokines in rat RBL-2H3 cells induced by immunoglobulin E-antigen complexes, resulting in the antagonism of passive cutaneous anaphylaxis [18]. In mouse RAW 264.7 cells, evodiamine inhibited lipopolysaccharide-induced prostaglandin E_2_ synthesis at concentrations of 1–10 mM [19]. Human A549 cells treated with evodiamine produced less monocyte chemoattractant protein-1 (MCP-1) after exposure to H1N1 and recruited fewer macrophages toward chemokine (C-C motif) ligand 5 (CCL5) and MCP-1 [20]. A concentration-dependent inverse relationship has been observed between evodiamine and endothelial cell cyclooxygenase 2 (COX-2) and nitric oxide synthase (iNOS) expression under hypoxic conditions [21]. The release of calcitonin gene-related peptides by evodiamine inhibits guinea pig heart anaphylaxis induced by bovine albumin [22]. According to Pearce et al., evodiamine is a new class of agonist of the rat transient receptor potential subfamily V member 1 (TRPV1) [23]. However, its potency is about 3–19-fold lower than that of capsaicin. In vivo studies have shown that evodiamine inhibits ovalbumin (OVA)-induced allergic asthma [24]. However, the efficacy of evodiamine in atopic dermatitis has not yet been examined. This study aimed to explore whether evodiamine acts as an anti-atopic agent and to investigate the mechanism of action of evodiamine in a dinitrochlorobenzene-induced atopic dermatitis-like mouse model.

## 2. Results

### 2.1. Evodiamine Suppresses Atopic Dermatitis Symptoms in the Ears of Mice

**Figure 1:**
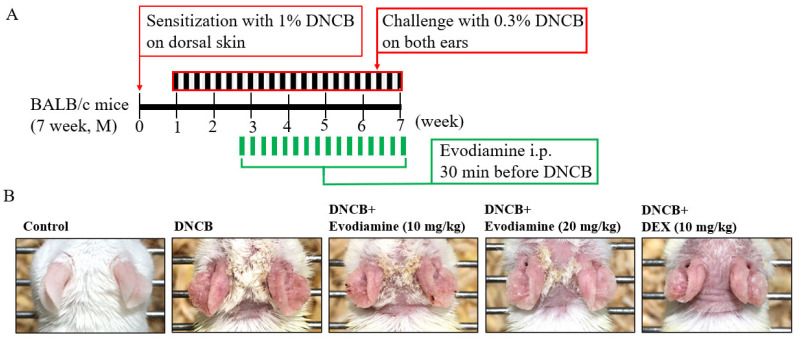
Effect of evodiamine on DNCB-induced atopic dermatitis in the ears. Evaluation of the effect of evodiamine on atopic dermatitis induced by DNCB in ears. (**A**) An outline of the protocol for induction of atopic dermatitis and treatment with evodiamine. On day 0, DNCB was applied to the skin to sensitize it. Following repeated DNCB challenges on days 7–48, atopic dermatitis-like phenotypes were induced. In addition to DNCB, vehicles were applied topically to BALB/c mice, while evodiamine and DEX were injected intraperitoneally 30 min before DNCB exposure (*n* = 5). (**B**) A view of the ear in its entirety.

According to Evodia fruits, evodiamine is a major alkaloid that exerts anti-inflammatory effects on mast cells, macrophages, and lung epithelial cells; the cytokines tumor necrosis factor-α (TNF-α) and IL-4 were suppressed in RBL-2H3 mast cells [18], prostaglandin E_2_ synthesis was inhibited in RAW 264.7 macrophages [19], and MCP-1 production was inhibited in lung epithelial cells A549 [20]. Evodiamine was hypothesized to be a therapeutic candidate for atopic dermatitis based on its anti-inflammatory effects, and we examined its effect on 2,4-dinitro-1-chloro-benzene (DNCB)-induced atopic dermatitis responses in mice. From days 7 to 48 after sensitization by DNCB intraperitoneal injection, DNCB were topically challenged every other day [25]. Evodiamine and DEX were administered by intraperitoneal injection 30 min before the DNCB challenge on day 19. Mice were sacrificed on day 49 (Figure 1A).

**Figure 2:**
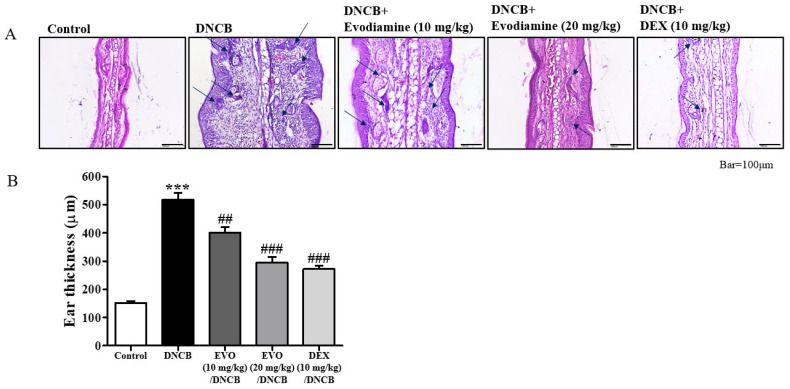
Effect of evodiamine on ear thickness. These are representative histological findings from cutaneous tissue sections taken on day 49. In order to stain the ear tissue, hematoxylin and eosin stain was applied (**A**). Blue arrows indicated immune cells. A comparison of the ear thickness between the groups was made in (**B**). Five mice were used for each group. Statistical significance: *** *p* < 0.001 vs. the vehicle-treated control group; ### *p* < 0.001, ## *p* < 0.01 vs. the DNCB-treated group. A magnification of 200× was used.

**Figure 3:**
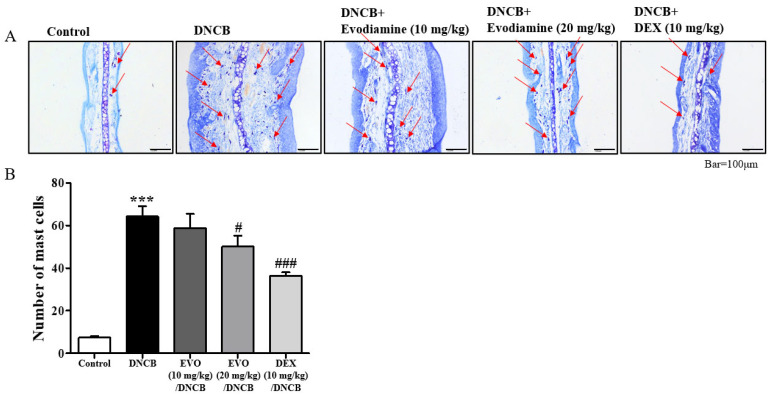
Effect of evodiamine on mast cell count in the ears. In order to identify mast cells, the skin was stained with toluidine blue O. (**A**) Histological findings on day 49 of representative cutaneous tissue sections. Mast cells are indicated with red arrows. (**B**) The number of mast cells in the ear tissues is shown in the histogram (*n* = 5). Statistical significance: *** *p* < 0.001 vs. the vehicle-treated control group; ### *p* < 0.001, # *p* < 0.05 vs. the DNCB-treated group. A magnification of 200× was used.

Scaling, erythema, and erosions were observed in the ears after exposure to DNCB (Figure 1B). Evodiamine-treated mice showed improvements in atopic dermatitis-like symptoms, whereas DEX-treated mice showed more significant improvements, especially swelling of the ears, a characteristic atopic dermatitis-like symptom. As shown in Figure 2A, cutaneous hyperplasia is clearly visible. Evodiamine and DEX significantly reduced DNCB-induced epidermal hyperplasia in response to DNCB (Figure 2A), which was measured as the thickness of the ear (Figure 2B). The suppressive effects of evodiamine (20 mg/kg) were equivalent to those of DEX (Figure 2B). In addition to acanthosis, an increase in epidermal thickness was observed by hematoxylin and eosin (H&E) staining, as well as exocytosis and increased immune cell accumulation (Figure 2A). By counting mast cells using toluidine blue O staining, evodiamine-treated atopic dermatitis mice displayed a significant suppression of immune cell accumulation (Figure 2A and Figure 3A).

In response to allergens, mast cells release pro-inflammatory cytokines and histamine, which contribute to atopic dermatitis symptoms. Toluidine blue O staining revealed mast cell infiltration of the dermis (Figure 3A). DNCB treatment resulted in a dramatic increase in mast cell numbers and hyperplasia (Figure 3A). In evodiamine-treated mice, the number of mast cells significantly decreased in a dose-dependent manner (Figure 3B). The number of mast cells was significantly suppressed in mice treated with DEX, similarly to that observed in mice treated with evodiamine (Figure 3B).

### 2.2. Evodiamine Suppresses Pro-Inflammatory Cytokine Expressions in the Ears of Mice

**Figure 4:**
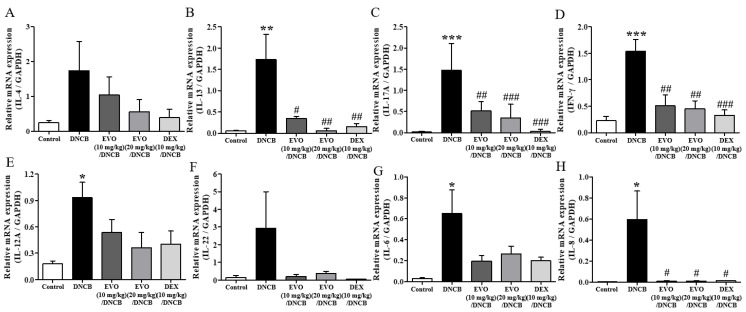
Effect of evodiamine on pro-inflammatory cytokine expression in the ears. Based on mouse ear tissue mRNA isolated from the ears of mice, qRT-PCR analysis of the Th2 cytokines IL-4 (**A**) and IL-13 (**B**) cytokines, Th17 cytokine, IL-17A (**C**), and Th1 cytokine, INF-γ (**D**) and IL-12A (**E**), Th22 cytokine, IL-22 (**F**), and chemokines IL-6 (**G**) and IL-8 (**H**) was conducted (*n* = 5). Statistical significance: *** *p* < 0.001, ** *p* < 0.01, * *p* < 0.05 vs. the vehicle-treated control group; ### *p* < 0.001, ## *p* < 0.01, # *p* < 0.05 vs. the DNCB-treated group. Normalization was also performed by comparing mRNA levels to GAPDH mRNA levels.

In atopic dermatitis pathogenesis, inflammatory cytokines of the Th2, Th17, Th1, and Th22 subclasses, such as IL-4, IL-13, IL-17A, INF-γ, IL-12A, and IL-22, play important roles [26,27,28]. In addition, the chemokines IL-6 and IL-8 attract inflammatory immune cells to the site of an atopic dermatitis [26,27,28]. There was an increase in cytokine and chemokine mRNA expression levels in the ear sections of atopic dermatitis-induced mice (Figure 4). In this study, both evodiamine and DEX significantly reduced the elevated mRNA levels of cytokines, particularly IL-13, IL-17A, IFN-γ, and IL-8 (Figure 4).

### 2.3. Evodiamine Suppresses DNCB-Induced Immune Responses in Lymph Nodes

**Figure 5:**
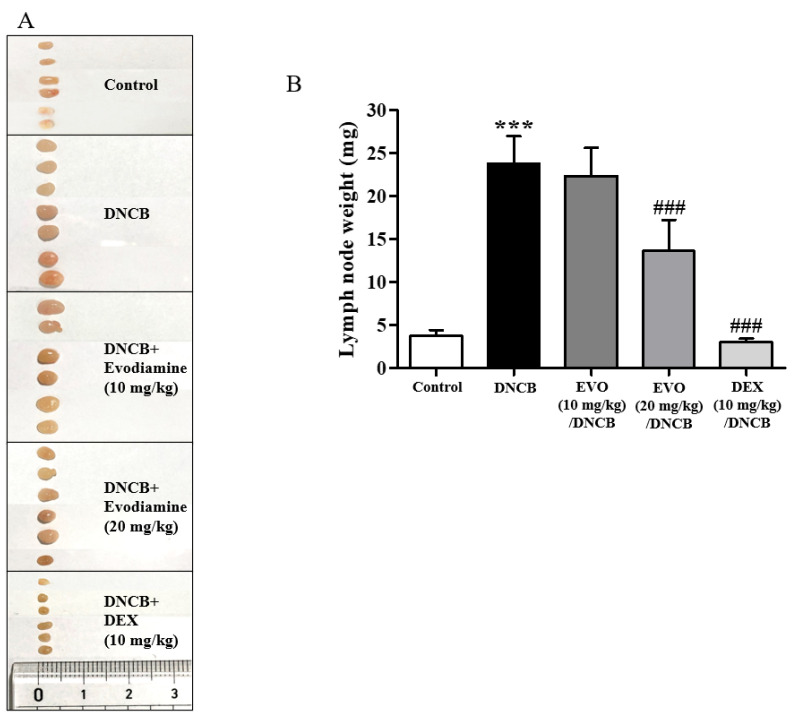
Effect of evodiamine on the size of lymph nodes. (**A**) Photographs were taken of the lymph nodes to measure their morphological changes. (**B**) A measurement of lymph node weight was also conducted (*n* = 5). Statistical significance: *** *p* < 0.001 vs. the vehicle-treated control group; ### *p* < 0.001 vs. the DNCB-treated group.

Next, the lymph nodes of the cervical region were examined for immune responses (Figure 5A). This response was significantly suppressed (Figure 5B). Significant inhibition was observed after treatment with 20 mg/kg evodiamine, but not as much as after DEX treatment (Figure 5B).

**Figure 6:**
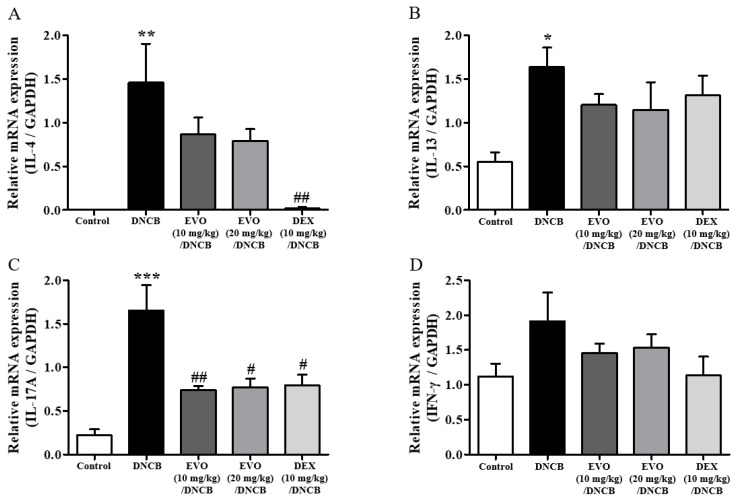
Effect of evodiamine on the expression of pro-inflammatory cytokines in lymph nodes. Based on mouse lymph node tissue mRNA isolated from the lymph nodes of mice, qRT-PCR analysis of the Th2 cytokines IL-4 (**A**) and IL-13 (**B**) cytokines, Th17 cytokine, IL-17A (**C**), and Th1 cytokine, IFN-γ (**D**) was conducted (*n* = 5). A normalization was also performed by comparing mRNA levels to GAPDH mRNA levels. Statistical significance: *** *p* < 0.001, ** *p* < 0.01, * *p* < 0.05 vs. the vehicle-treated control group; ## *p* < 0.01, # *p* < 0.05 vs. the DNCB-treated group.

Additionally, DNCB-induced atopic dermatitis mice exhibited significantly increased Th2 (IL-4 and IL-13), Th1 (IFN-γ), and Th17 (IL-17A) cytokine mRNA levels in the cervical lymph nodes (Figure 6A–D). The treatment efficacy of evodiamine was shown by the substantial suppression of the mRNA levels of cytokines, especially IL-17A (Figure 6C). In summary, the findings of the current study indicate that evodiamine reduces lymph node enlargement and the expression of inflammatory Th17 cytokines.

### 2.4. Evodiamine Suppresses Pro-Inflammatory Cytokine Expressions in the Ears of Mice

**Figure 7:**
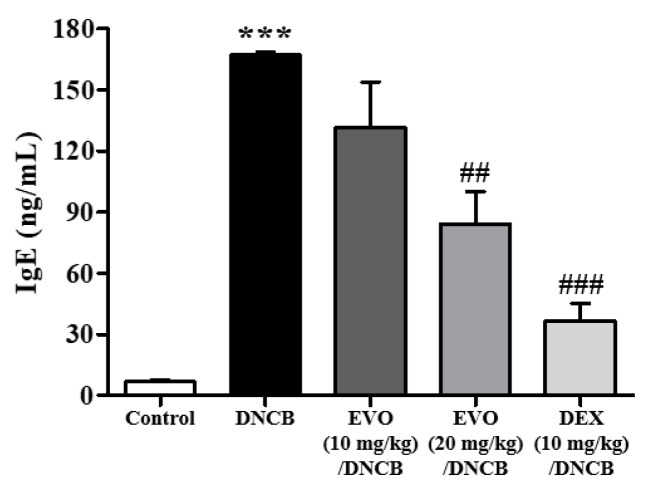
Effect of evodiamine on serum immunoglobulin E levels. Day 49 was the day on which serum was collected from the animals. An enzyme-linked immunosorbent assay was used to measure serum IgE levels (*n* = 5). Statistical significance: *** *p* < 0.001 vs. the vehicle-treated control group; ### *p* < 0.001, ## *p* < 0.01 vs. the DNCB-treated group.

IgE is a key player in the pathogenesis of atopic dermatitis, despite the considerable heterogeneity of the condition. A significant elevation in IgE levels is a key characteristic of atopic dermatitis and is associated with disease severity. Therefore, ELISA was used to test IgE levels in the serum obtained on day 49. The PBS group exhibited low serum IgE levels (Figure 7). However, the DNCB challenge can easily elevate IgE levels. DNCB plus evodiamine treatment significantly reduced IgE levels in a dose-dependent manner, but the efficacy of evodiamine was lower than that in the DEX-treated groups (Figure 7).

## 3. Discussion

A chemically induced animal model was used to determine how evodiamine affects atopic dermatitis, which is a chronic inflammatory skin condition. Based on previous studies, the doses of evodiamine used in this study were determined to be between 10 and 20 mg/kg. This is the first study to demonstrate that evodiamine is effective against atopic dermatitis, as indicated by the suppression of ear thickening, mast cell accumulation, lymph node enlargement, IgE levels in the serum, and Th1/Th2/Th17/Th22 cytokines and chemokines. It has previously been demonstrated that evodiamine (25 mM) suppresses passive cutaneous anaphylactic symptoms in mice and exerts inhibitory effects on RBL-2H3 mast cells in vitro [18]. By injecting exogenous IgE directly into the skin, the model delivered antigen-specific IgE [29]. Therefore, the effect of a compound on the suppression of anaphylactic cutaneous reactions can be evaluated without active immune responses from antigen sensitization. Instead, the present atopic dermatitis model was induced by sensitization and challenge with antigens, resulting in more relevant conditions similar to in vivo atopic dermatitis. Similarly, Wang et al. [24] found that evodiamine (40 and 80 mg/kg) protected Sprague-Dawley rats from ovalbumin-induced asthma. In a rat model of allergic asthma, evodiamine inhibited allergic responses, which is in agreement with the results of our current study [24]. It was possible to observe the entire immune response to ovalbumin-induced asthma due to both antigen sensitization and challenge. Evodiamine reduced the thickness of the airway wall of the small bronchioles in the asthma group [24], which was similar to the reduction in the thickness of the ear and lymph nodes in this study. It was found that evodiamine caused downregulation of toll-like receptor-4, MyD88, nuclear factor κ-light-chain-enhancer of activated B cells (NF-κB), and high mobility group box 1 (HMGB1) mRNA in lung tissues [24]. It would be worth investigating whether evodiamine suppresses toll-like receptor 4 (TLR-4), myeloid differentiation primary response gene 88 (Myd88), NF-κB, and HMGB1 mRNA expression in the skin.

A primary mode of action of evodiamine may be its anti-inflammatory effect, as evodiamine was found to have anti-inflammatory properties in mast cells, macrophages, and lung epithelial cells [18,19,20]. Previous studies have shown that evodiamine inhibits cytokine production in RBL-2H3 mast cells [18], prostaglandin E_2_ synthesis in RAW 264.7 macrophages [19], and the production of chemotactic MCP-1 in A549 lung epithelial cells [20]. In the bronchoalveolar lavage fluid and lung tissue, evodiamine inhibits IL-4, IL-13, and IL-17 [24]. As a result of evodiamine treatment, TNF-α and IL-4 levels were suppressed in RBL-2H3 mast cells [18]. Treatment of rutaecarpine, another indoloquinazoline alkaloid from the fruits of *Evodia rutaecarpa*, reduced plasma levels of IL-4 and IgE but enhanced plasma IFN-γ levels, which is contrasting to our results [30]. Evodiamine suppressed expression of pro-inflammatory COX-2 and iNOS as well as prostaglandin E_2_ release under hypoxic conditions [21] and suppressed cardiac anaphylaxis in isolated guinea-pig hearts [22].

This study indicated that evodiamine may have anti-inflammatory effects on several immune cells that affect atopic dermatitis, as in allergic asthma models. Because of its high binding affinity to TLR-4, evodiamine can be considered a molecular target of TLR-4, since molecular docking studies of evodiamine with TLR-4 have shown its drug-binding affinity [24]. Although TRPV1 is also considered a potential target of evodiamine for pain perception [31], it is unlikely to be responsible for its anti-atopic dermatitis effects.

In addition to evodiamine, rutaecarpine also has anti-inflammatory properties [19]. A previous study showed that rutaecarpine inhibited the COX-2-dependent generation of prostaglandin D_2_ in bone marrow-derived mast cells and reduced carrageenan-induced paw swelling [32]. Rutaecarpine treatment promoted macrophage immune training activators, including fos-related antigen 2 (FOSL2), SWI/SNF-related, matrix-associated, actin-dependent chromatin regulator subfamily a, member 4 (SMARCA4), and signal transducer and activator of transcription 3 (STAT3) [33], resulting in anti-inflammatory effects. Furthermore, rutaecarpine reduced the symptoms of atopic dermatitis in mice carrying the NC/Nga genotype [30].

We demonstrated the anti-atopic dermatitis effects of evodiamine in a murine model, demonstrating that the effectiveness of evodiamine was caused by the inhibition of mast cell accumulation in the skin, along with the inhibition of pro-inflammatory cytokines (IL-17A, IL-4, IL-13, IFN-*γ*, and IL-12A) and chemokines (IL-8 and IL-6) in the lymph nodes and epidermis. Based on the results of this study, evodiamine appears to have the potential to treat atopic dermatitis.

## 4. Materials and Methods

### 4.1. Chemicals

The 2,4-dinitro-1-chloro-benzene (DNCB) and evodiamine (Cat No. E3531, purity: ≥98% in HPLC) were purchased from Sigma-Aldrich (St. Louis, MO, USA).

### 4.2. Mouse Strain

We purchased male, 7-week-old BALB/c mice from Daehan Biolink (Seoul, Republic of Korea). Animal protocols were reviewed and approved by the Kyung Hee University-Institutional Animal Care Committee (KHSASP-23-012). The mice were housed in two per plastic cage under conditions of temperature at 22–24 °C, humidity at 60 ± 5%, and alternating light/dark cycles. We provided standard laboratory chow and water ad libitum.

### 4.3. Induction of DNCB-Induced Atopic Dermatitis in BALB/c Mice

Eight-week-old BALB/c mice were randomized into five groups (*n* = 5): a vehicle-treated control group, a DNCB-treated group, a DNCB-treated and evodiamine (10 mg/kg)-administered group, a DNCB-treated and evodiamine (20 mg/kg)-administered group, and a DNCB-treated and dexamethasone (DEX, 10 mg/kg)-administered group. To induce atopic dermatitis-like symptoms, we used DNCB as previously described [34]. Dorsal skin was shaved, and 300 µL of 1% DNCB in acetone/olive (3:1) was spread to the dorsal skin on day 0, which is sensitization. On day 7, 200 µL of 0.3% DNCB was applied to both ears every other day for up to 48 days. From day 19 until completion of the experiment, the evodiamine/DNCB-treated group was administered evodiamine (10 or 20 mg/kg body weight) by intraperitoneal injection 30 min prior to the challenge. The mice were sacrificed on day 49.

### 4.4. Total Immunoglobulin E (IgE) Levels in Serum

Total serum IgE levels were assessed using an ELISA kit (eBioscience, San Diego, CA, USA). In brief, 96-well plates (NUNC, Penfield, NY, USA) were coated with eBioscience’s IgE capture antibody (88-50460-88, San Diego, CA, USA) and incubated overnight at 4 °C. After the washing, the plates were incubated at room temperature for 2 h with blocking buffer. Standard IgE was serially diluted and added to the appropriate wells to generate a calibration curve. Serum samples were added to each well. The plates were incubated at room temperature for 2 h on a shaker, followed by two washes. Each well was incubated with a biotinylated antibody designed to detect IgE (cat. 88–50460–88, eBioscience, San Diego, CA, USA). The plates were shaken for 1 h at room temperature. Each well was treated with avidin-horseradish peroxidase (HRP) after four washes. The incubation was performed in a shaker at room temperature for 30 min. After washing the plates four times, the substrate solution was applied, and the plates were incubated for 15 min at room temperature. The absorbance was measured at 450 nm after the addition of the stop solution.

### 4.5. Mast Cell Count in the Skin

Ear sections from mice in different experimental groups were analyzed after sacrifice on day 49. A 10% formalin fixative was used along with a 30% sucrose solution to dehydrate the ears, and an O.C.T. compound was used to embed the ears. To visualize mast cells in the skin, eight micrometer sections were stained with toluidine blue O (cat. T3260, Sigma-Aldrich, St. Louis, MO, USA). In each group, the tissues of five mice were examined. After removing the O.C.T. compound from the sections, the toluidine blue O reagent was added to the sections and soaked for 2 min. The sections were then rinsed, dehydrated, and mounted on coverslips. The number of mast cells was counted by analyzing photographs taken after toluidine blue O staining. Toluidine blue O staining was used to detect mast cells. The mast cells were counted twice in 50 optical fields, and the average was used [34].

### 4.6. Histologic Analysis and Measurement of Ear Thickness

The ear skin of each mouse was excised on day 49. The ears from different groups were fixed with neutral-buffered formalin (10%), dehydrated in a sucrose solution (30%), and embedded in the O.C.T. compound. The sections (8 µm) were stained with hematoxylin and eosin (H&E). For H&E staining, the sections were washed under running tap water for 5 min and counterstained with a hematoxylin solution for 90 s. After washing under running tap water, the sections were stained with eosin solution for 10 s, rinsed, dehydrated, and coverslipped with Permount mounting medium. Ear thickness was determined using H&E-stained photomicrographs. The average value was calculated by measuring five measurements per mouse. ImageJ software (version 1.54g, National Institutes of Health, Bethesda, MD, USA) was used to assess skin thickness by measuring the distance.

### 4.7. Quantitative Real-Time PCR

The expression levels of inflammatory markers in the ears of the mice were measured using qRT-PCR. Total RNA in the lymph nodes and skin tissue was isolated using Trizol^®^ (Invitrogen, Waltham, MA, USA). The RNA was reverse transcribed into cDNA using MMLV reverse transcriptase (Promega, Madison, WI, USA). Thunderbird Next SYBR qPCR Mix was used for qRT-PCR on a CFX Connect Real-Time System (Bio-Rad, Hercules, CA, USA). The PCR program consisted of a cycle at 95 °C for 4 min, 40 cycles at 95 °C for 30 s, 57 °C for 30 s, and the last at 95 °C for 30 s. With the help of CFX Maestro Software version 2.3 (Bio-Rad Laboratories, Hercules, CA, USA), the obtained data were analyzed using the 2^−∆∆Ct^ method. The primer sequences are listed in Table 1. The results were normalized to GAPDH gene expression levels [35].

### 4.8. Statistics

We expressed the results as the mean ± standard error of the mean (SEM) of five determinations for each animal group. The statistical significance of differences was determined by analysis of variance (ANOVA) and Tukey’s multiple comparison test. GraphPad Prism software version 5 (GraphPad Software, Inc., La Jolla, CA, USA) was used for data analysis. Statistical significance was accepted for *p* < 0.05: * *p* < 0.05, ** *p* < 0.01, and *** *p* < 0.001, vs. the vehicle-treated control group; # *p* < 0.05, ## *p* < 0.01, and ### *p* < 0.001 vs. the DNCB-treated group.

## Figures and Tables

**Table 1:** Quantitative real-time PCR primers.

Mouse Primers		Sequence
*Il-6*	forward	5′-TTC TTG GGA CTG ATG CTG GT-3′
	reverse	5′-CTG TGA AGT CTC CTC TCC GG-3′
*Il-8*	forward	5′-AAC TCC TTG GTG ATG CTG GT-3′
	reverse	5′-CCA GGT TCA GCA GGT AGA CA-3′
*Il-12A*	forward	5′-GAA GCT CTG CAT CCT GCT TC-3′
	reverse	5′-CAG ATA GCC CAT CAC CCT GT-3′
*IFN-γ*	forward	5′-CAC GGC ACA GTC ATT GAA AG-3′
	reverse	5′-GTC ACC ATC CTT TTG CCA GT-3′
*Il-4*	forward	5′-TCT CGA ATG TAC CAG GAG CC-3′
	reverse	5′-CCT TCT CCT GTG ACC TCG TT-3′
*Il-13*	forward	5′-GCA GCA TGG TAT GGA GTG TG-3′
	reverse	5′-AGG CCA TGC AAT ATC CTC TG-3′
*Il-22*	forward	5′-GTC AAC CGC ACC TTT ATG CT-3′
	reverse	5′-GTT GAG CAC CTG CTT CAT CA-3′
*Il-17A*	forward	5′-TCC AGC AAG AGA TCC TGG TC-3′
	reverse	5′-AGC ATC TTC TCG ACC CTG AA-3′
*Gapdh*	forward	5′-AGA ACA TCA TCC CTG CAT CC-3′
	reverse	5′-CAC ATT GGG GGT AGG AAC AC-3′

## Data Availability

Data are contained within the article.
